# Prefrontal Markers and Cognitive Performance Are Dissociated during Progressive Dopamine Lesion

**DOI:** 10.1371/journal.pbio.1002576

**Published:** 2016-11-08

**Authors:** Charles R. E. Wilson, Julien Vezoli, Frederic M. Stoll, Maïlys C. M. Faraut, Vincent Leviel, Kenneth Knoblauch, Emmanuel Procyk

**Affiliations:** University of Lyon, Université Claude Bernard Lyon 1, INSERM, Stem Cell and Brain Research Institute U1208, Lyon, France; University of Minnesota, UNITED STATES

## Abstract

Dopamine is thought to directly influence the neurophysiological mechanisms of both performance monitoring and cognitive control—two processes that are critically linked in the production of adapted behaviour. Changing dopamine levels are also thought to induce cognitive changes in several neurological and psychiatric conditions. But the working model of this system as a whole remains untested. Specifically, although many researchers assume that changing dopamine levels modify neurophysiological mechanisms and their markers in frontal cortex, and that this in turn leads to cognitive changes, this causal chain needs to be verified. Using longitudinal recordings of frontal neurophysiological markers over many months during progressive dopaminergic lesion in non-human primates, we provide data that fail to support a simple interaction between dopamine, frontal function, and cognition. Feedback potentials, which are performance-monitoring signals sometimes thought to drive successful control, ceased to differentiate feedback valence at the end of the lesion, just before clinical motor threshold. In contrast, cognitive control performance and beta oscillatory markers of cognitive control were unimpaired by the lesion. The differing dynamics of these measures throughout a dopamine lesion suggests they are not all driven by dopamine in the same way. These dynamics also demonstrate that a complex non-linear set of mechanisms is engaged in the brain in response to a progressive dopamine lesion. These results question the direct causal chain from dopamine to frontal physiology and on to cognition. They imply that biomarkers of cognitive functions are not directly predictive of dopamine loss.

## Abstract 2

Neural recordings during dopaminergic depletion shed doubt on our current understanding of the direct link between frontal dopaminergic inputs, frontal neurophysiology, and cognitive performance.

## Author Summary

To successfully complete a task, we need to monitor our performance. If performance drops, we need to change our behaviour. We do this by adjusting cognitive control, an ensemble of processes through which behaviour is adapted to suit the task. In this study, we first used chronic recordings in the frontal lobe of macaque monkeys to characterise neurophysiological markers that reflect these processes: a brain potential reflecting performance monitoring and a sustained oscillatory signal reflecting cognitive control. It has been suggested that cognitive control, performance monitoring, and their neurophysiological markers are under the influence of dopamine. To understand how the input of dopamine is critical, we followed changes in the markers and performance during slow dopaminergic depletion. This protocol doubles up as a study of the early phase of Parkinson’s disease, when dopaminergic cells are dying but motor symptoms have yet to emerge. Whilst the performance monitoring potential attenuated at the end of the depletion, the performance itself did not. The oscillatory signals showed only subtle changes in comparison, despite the depletion. Together these results bring into question the simple idea that dopamine directly modulates frontal cortex, which in turn directly modulates cognition. We consider how the brain may compensate for a dopamine lesion, and whether the markers measure what we think they do. Our results question a current idea that neurophysiological markers can be directly used to predict dopamine loss in patients with conditions like Parkinson’s disease.

## Introduction

Successful and adaptive completion of cognitive tasks requires tight integration between performance monitoring [1,2], which provides information about task outcomes, and cognitive control [3], which drives behavioural adaptation as necessary. These systems are associated with neurophysiological markers in the frontal lobes that are modulated by motivation [4].

Error- and feedback-related potentials (error-related negativity [ERN] and feedback potentials [FRPs]) recorded over the medial part of the frontal lobe in electroencephalography (EEG) [5–7], electrocorticography (ECoG) [8], and local field potential (LFP) [9] differentiate outcome valences. These performance-monitoring signals are in many cases generated in midcingulate cortex (MCC [10,11]). These signals appear to provide information about the value of the feedback in terms of behavioural adaptation [12,13], be it for directly driving adaptation on subsequent trials [14] or for motivating more extended behaviours beyond simple trial-to-trial adaptation [15–17].

A second key constituent of this integrated system implements the chosen level of control and is associated with lateral prefrontal cortex (PFC). Control implementation is signaled, for example, in modification of classical working memory delay activity [18], and has been linked to PFC beta oscillatory power. In frontal cortex, beta oscillations are implicated in top-down control of behaviour in cognitively engaging tasks [19–22], whilst also altering within-session to reflect attentional effort on the task [23].

Dopamine (DA) is proposed to have a critical role in regulating these systems and the related behaviour [24], and theoretical and computational models support a link between dopamine dysfunction and a range of cognitive symptoms in neurological disorders [25,26]. A working model has been proposed that directly links DA to both performance monitoring and cognitive control in frontal cortex. First, the mesocortical dopaminergic projections are thought to provide a prediction error signal that regulates performance monitoring functions implemented in MCC [10]. However, causal proof for this relation remains sparse [8], dopamine antagonist interventions have variable effects on behavioural outcomes [27,28], and so the functional significance of this link is debated [29]. Second, dopamine has been directly or indirectly linked to neurophysiological prefrontal mechanisms of cognitive control [30,31], working memory [32–34], and motivation [35]. If dopamine has a clear role in these mechanisms, it should be revealed in diseases with an altered dopaminergic system [2], and dopamine loss should be related to the relevant behavioural and cognitive deficits. But it remains unclear whether this relationship is as direct and simple as the model proposes [36]. There is evidence for ERN modification in Huntington’s disease [37] and schizophrenia [38], though the extent to which these effects are a result of dopaminergic changes is unclear. The dopamine system is implicated in impairments of cognitive control and motivation in Parkinson’s disease (PD) [39], yet neurophysiological studies of PD patients provide only mixed evidence for and against the modification of ERN [40–43], and FRP [44].

Proper testing of this working model requires a systemic approach combining dopaminergic modulation, neurophysiology of frontal mechanisms, and related cognitive control performance. This approach is absent from the literature, and yet this test is a mandatory step to understanding dopamine-neurophysiology-cognition links, the role of dopamine in driving frontal functions, and how to target treatments of the relevant conditions.

Here, we reveal the dynamic of performance monitoring, cognitive control, and their neurophysiological markers during a progressive lesion of the dopamine system. In particular, we use a test of cognitive control [18] known to share direct prefrontal neuronal mechanisms with dopamine-sensitive working memory [45,46]. Contrary to the working model, we found dissociations between evoked markers of performance monitoring (feedback-related potentials) and performance on the task itself, and between induced markers of cognitive control and motivation (frontal beta oscillations). Our data, therefore, argue against a simple interaction between dopamine and frontal functions.

## Results

We tested the effect of progressive dopaminergic loss on electrophysiological markers of performance monitoring and cognitive control, using chronic ECoG recordings in monkeys. Signals were acquired over extended periods (Monkeys R and S: 141 and 213 d, respectively) whilst inducing a very slow progressive dopaminergic lesion with the neurotoxin 1-methyl-4-phenyl-1,2,3,6-tetrahydropyridine (MPTP). We compared this with a substantial pre-lesion baseline period (referred to as “BL period” throughout: Monkeys R and S: 46 and 55 d, respectively) in a within-monkey design.

### Task and BL Period Behaviour

**Fig 1:**
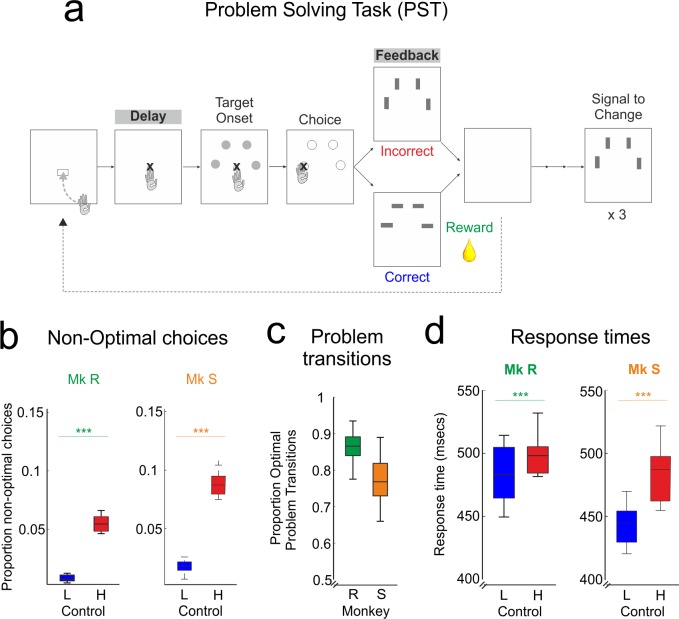
Task and BL behaviour. Monkeys perform the PST efficiently prior to MPTP treatment, demonstrating understanding of the task and cognitive control. **A.** Problem-solving task PST4, with task epochs titled from left to right. Monkeys sought, by trial and error using feedback, which of the four grey targets was rewarded, and then repeated this correct choice three times to complete a problem. Delay and feedback epochs for neurophysiological data are highlighted **B**. Proportion of non-optimal choices (repeated incorrect choices in SEA, errors in REP). Monkeys are performing the task well, and the majority of non-optimal choices were on high-control trials. **C**. Proportion of optimal problem transitions. Monkeys were near optimal, showing clear use of the STC. **D**. Reaction times on low and high control trials. Mk: Monkey (Monkey R and Monkey S). L: Low control trials; H: High control trials. Raw data for this and all following figures are freely available to download via the link in the Data Availability Statement. The folder in this download contains a readme file describing the contents.

Two monkeys learned the problem-solving task (PST, Fig 1A), [18], a test of cognitive control during which they had to search (SEA phase) by trial and error using feedback amongst four visually identical stimuli to find the location rewarded by juice. Once monkeys had found the rewarded location, they could repeat that rewarded choice three times (repetition phase, REP), before a signal to change (STC) informed them that the rewarded location had been re-randomized. Previous research has shown that the PST induces high or low cognitive control on different trials [8,12,18,23]. Low control is sufficient on any repetition trial after correct feedback, as the monkey simply has to repeat the previous choice. High control is required when the outcome of the previous trial necessitates a behavioural adaptation—notably, in three cases, after an incorrect feedback, after an STC directing a new SEA phase, and after the monkey makes a break in fixation or touch. This task is a well-established test of cognitive control with well-established neural correlates, notably delay activity in PFC comparable to working memory tasks [18] and feedback responses sensitive to dopamine [8]. As such, it allows us to probe control in terms of both performance monitoring and control implementation.

In the BL period, monkeys were tested for at least 10 weeks with sham injections to establish baseline performance and neurophysiology. During this time, monkeys’ performance approached optimality. Their level of non-optimal responses (repeated choices in SEA, errors in REP; see Materials and Methods) was low, at less than 10% (Fig 1B), and they showed, as expected, lower levels of non-optimal choice after low-control than high-control trials (ANOVA, Monkey R: F_(1, 26189)_ = 638, *p* < 0.0001; Monkey S: F_(1,13449)_ = 563, *p* < 0.0001, Fig 1B). Furthermore, they showed clear and responsive transition between REP and SEA phases, taking into account the STC and changing their choice on the following trial (Fig 1C). Response times (RTs) over choices also reflected these levels of control, with faster RTs on low-control trials after a correct response (ANOVA, Monkey R: F_(1,26189)_ = 59, *p* < 0.0001; Monkey S: F_(1,13449)_ = 236, *p* < 0.0001, Fig 1D). A similar effect is present in the reaction times, more commonly used in human experiments (S2 Fig). Together, these behavioural data demonstrate that monkeys are able to apply cognitive control to search for and then exploit reward possibilities, as previously shown in this task [12].

### Frontal Neurophysiology in BL Period

Monkeys were implanted with grids of 22 and 31 trans-cranial ECoG electrodes covering the frontal lobes (Monkeys R and S respectively, S1B Fig). We aligned ECoG signals to analyze individual trials during the delay epoch (Fig 1A), when the monkey awaits the start of the trial, and the feedback epoch.

**Fig 2:**
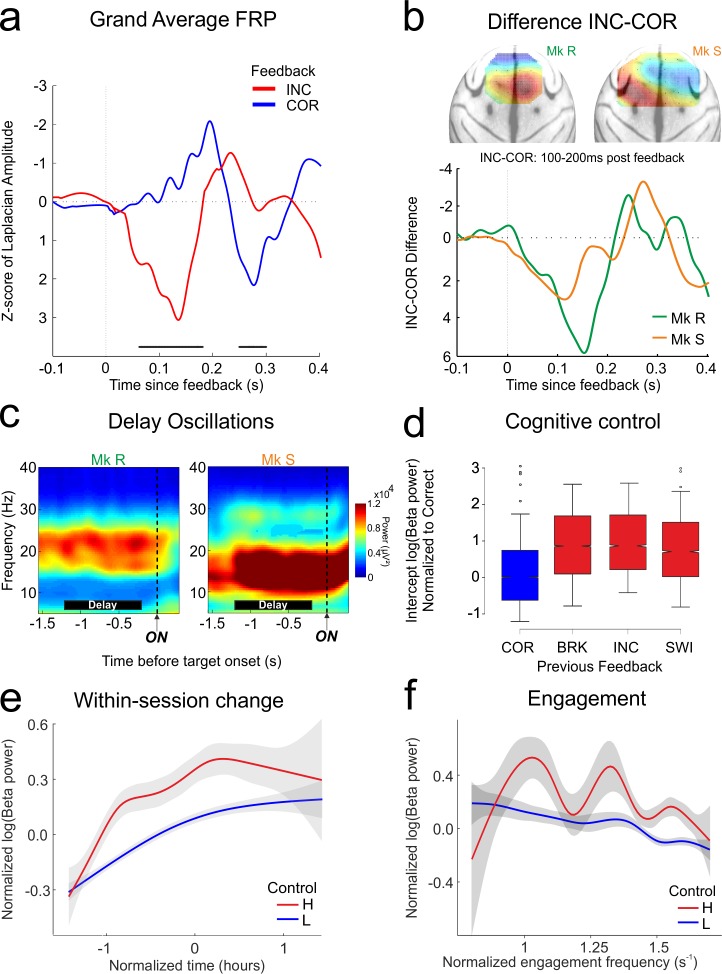
Frontal neurophysiology in BL. Performance monitoring, cognitive control, and motivation are represented in neurophysiological markers within prefrontal cortex prior to the onset of MPTP treatment. **A**. Feedback potential grand average combining both monkeys, aligned to feedback presentation, showing clear differentiation of correct (COR) from incorrect (INC) feedback. Black bars: permutation test *p* < 0.01 between correct and incorrect. **B**. Difference potentials INC-COR for the FRPs, and spatial representations of the surface Laplacian of these potentials projected onto a dorsal view of a standard macaque brain. The peak difference (red) can be seen over the contralateral hemisphere to the working arm of the monkey (Monkey R left-handed, Monkey S right-handed). **C**. Time frequency representations of the delay epoch (black bars) aligned to stimulus onset (ON). **D–F**: Properties of delay beta power in BL as revealed by linear mixed effect model selection. For the figures, data are normalized, then combined for the two monkeys. **D**. Modulation of delay beta power by the outcome of the previous trial. Power is increased when the previous outcome instructs application of higher cognitive control. Red bars, high control conditions: BRK: break fixation/touch, INC: incorrect choice, SWI: problem switch after STC. This is compared to blue bar, low control condition: COR: correct choice. Normalization is to the COR feedback condition. **E**. Within-session increase in beta power. There is no significant interaction between this factor and cognitive control, as revealed by an increase in beta for both low and high control trials. **F**. Reduction in beta power with increasing engagement frequency of the monkey. Conventions as in previous figure.

In BL, a medial frontal evoked response after feedback differentiated correct (COR) from incorrect (INC) feedback, with a significant response difference between 50 and 200 ms (grand average waveforms in Fig 2A, permutation test, *p* < 0.001 in both monkeys). This FRP therefore reflects critical information that might be required to adapt behaviour in the following trial, as best indexed by the difference curve shown in Fig 2B, a standard measure in the literature [47]. The FRP signal was highly stable over sessions, as we have previously demonstrated [8]. The surface Laplacian FRP is located relatively medially over prefrontal cortex, with a maximum contralateral to the working arm of each monkey (Monkey R left handed, Monkey S right handed). We and others have previously reported this marker in monkeys [8] and humans [5]. It is thought to arise from a source in the MCC [11] and to be sensitive to dopaminergic modulations [8,10].

The contrast INC–COR can reveal effects of feedback valence and/or feedback expectancy. Although the task is not perfectly designed to dissociate the two because monkeys are making free choices, we can provide evidence for one or the other by focusing on trials from the SEA phase. This includes INC and first correct (CO1) trials from each problem, excluding repeated COR feedback, for which the monkey has a higher expectation than CO1. In SEA, the probabilities of observing negative or positive feedback (INC and CO1) are roughly equivalent on average (S3B Fig, Monkey R: p(INC) = 0.46, 95% CI [0.4, 0.51]; Monkey S: p(INC) = 0.61, 95% CI [0.57, 0.65]; p(CO1) is the complementary in each case). S3A Fig shows that the difference curve is maintained in BL when considering the contrast INC-CO1. This suggests, therefore, that it is the valence of the feedback that mainly drives the observed FRP, rather than the expectancy of receiving each type of feedback.

During the delay epoch at the start of a trial, the monkey can prepare the upcoming choice based on previous choices and outcomes. Both monkeys showed strong induced oscillations in the beta band (15–30 Hz) throughout the delay (Fig 2C). We analysed the band of beta power that we had previously identified as being modulated by the cognitive elements of the task in each monkey [23] (see also Materials and Methods). The absolute value of delay beta power varies in a trial-by-trial manner with a number of factors. We used linear mixed-effects modeling [48] to reveal the contributions of these factors and account for the repeated measures nature of our design [23]. Importantly, here and throughout the study, we selected a linear mixed-effects model to describe the data through a model selection procedure. The process and selected model are presented in detail in the Materials and Methods. All models discussed herein contain only behavioural factors that have survived model selection. A number of potential factors notably did not survive model selection, including response times and optimality of choice (in each case, likelihood ratio test between nested models, *p* > 0.05). Note that the term “beta” refers to power of high beta oscillations, and never to any form of model beta (i.e., estimates).

The effect of cognitive control requirements was strong and consistent: any outcome that required the monkey to adapt behaviour—incorrect feedback, STC, or breaks—led to increased beta power during delay in the following trial, when compared to a positive outcome (“correct”) that simply led to a repetition of the previous choice (Fig 2D, Wald conditioned F test: Monkey R: F_(2,22790)_ = 370, *p* < 0.0001; Monkey S: F_(2,11312)_ = 39, *p* < 0.0001). In addition to cognitive control, two factors potentially related to motivation had significant impact on beta power. First, beta power significantly increased with time “within-session”—that is, in a given session, power correlated with the time the monkey had spent continuously working (Fig 2E, Wald conditioned F test: Monkey R: F_(1,22790)_ = 209, *p* < 0.0001; Monkey S: F_(1,11312)_ = 22, *p* < 0.0001). We have previously linked this within-session change to an increase of attentional effort of the monkey during sustained work, not least because the power increase is “reset” by a voluntary pause in work [23]. In that study we also showed that this attentional effort effect is independent of cognitive control. Second, the frequency with which the monkey engaged trials had a smaller but significant effect on beta power (Fig 2F, Wald conditioned F test: Monkey R: F_(1,22790)_ = 12, *p* = 0.0004; Monkey S: F_(1,11312)_ = 5.4, *p* = 0.02). Engagement is potentially a measure of the motivation for the task, although its interpretation is not unambiguous. The interaction between measures of motivation and cognitive control is a significant subject of interest (and confusion) in the field [4]. Here, trial-by-trial engagement and attentional effort both influenced beta power, but there were no interactions between these effects (likelihood ratio test between nested models, Monkey R: *p* = 0.23; Monkey S: *p* = 0.57), nor did they interact with cognitive control (same test, *p* > 0.15 in each case). This clearly suggests three separable drivers of beta power.

Hence, data presented in Figs 1 and 2 reveal that during the BL period, monkeys were performing the PST near optimally using performance monitoring and cognitive control, and that these processes are reflected in stable neurophysiological measures in frontal cortex by FRPs and beta oscillatory power.

### MPTP Protocol and Behaviour

**Fig 3:**
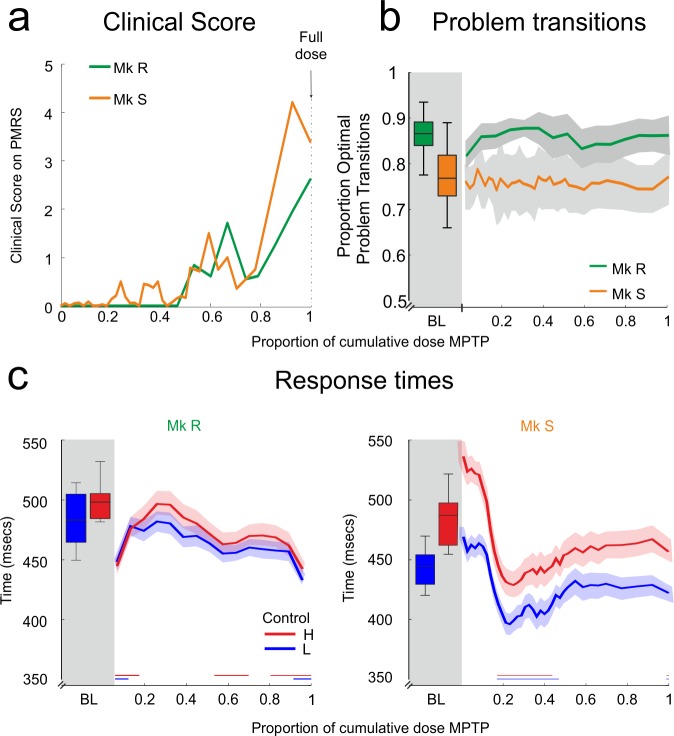
MPTP protocol and behaviour. MPTP lesion induces sub-threshold motor symptoms in the home-cage, showing that the lesion is occurring, but motor parameters on the task are not slowed. **A**. Motor symptom score for each monkey on the PMRS motor scale measured daily in the home-cage and averaged for each dose. In this and the following figures, MPTP is presented as a proportion of the full dose of MPTP received (see Materials and Methods). **B**. Proportion of optimal problem transitions over MPTP treatment. Monkeys continue to make the same level of optimal transitions as in the BL period. **C.** Response times in the PST task. Neither monkey shows a slowing of response in the task, but some speeding is present in both monkeys. Conventions as in previous figures.

Monkeys then received doses of 0.2 mg/kg of MPTP, a dose well established in progressive protocols [49,50]. MPTP injections were given at most once per week—significantly less frequently than most other studies. The protocol was designed to induce very slow degeneration whilst permitting concurrent recordings with sufficient task performance (see Materials and Methods). The protocol was long (Monkey R: 33 weeks; Monkey S: 56 weeks) so that gradual emergence of neural changes could be observed. Treatment continued until monkeys obtained a “significantly symptomatic” score of 5 on the Parkinsonian Monkey Rating Scale (PMRS) [50]. Monkeys therefore remained below this significantly symptomatic level throughout the MPTP period (Fig 3A), and the final period during which monkeys worked before attaining this level of symptoms is referred to as the “full dose.” Whilst the total cumulative dose was different, the pattern of symptomology across treatment was similar between the two monkeys (Fig 3A). This pattern is consistent with slow emergence of a dopaminergic lesion with MPTP, in line with other progressive protocols [49–51]. Note that the PMRS motor scale scoring is included for evaluation of the Parkinsonian state as applied in the literature. Our aim is to contrast it with changes in frontal neurophysiology and cognition, and it is not intended as an assessment of these latter measures.

PMRS scoring acted as the principle measure of lesion progress and determined cessation of the protocol. The use of a progressive MPTP protocol in conjunction with motor scoring is well established in the literature [49,51]. Monkeys showing full motor symptoms following MPTP treatment already have significant loss of nigral dopaminergic cells [52], and monkeys brought to a motor symptomatic state who subsequently recover nevertheless show reduced tyrosine hydroxylase (TH) cell labeling in the mesencephalon [51]. Previous work in our laboratory has measured the binding potential of the selective dopamine active transporter (DAT) radiotracer [^11^C]PE2I in monkeys in a progressive MPTP protocol [53]. The use of this tracer is also established in patients with Parkinson’s disease [54]. We showed that DAT binding is increased in the early phases of a progressive MPTP lesion, returning to baseline levels around the onset of symptoms and then dropping as motor symptoms become persistent. The final strong motor symptomatic phase is associated with significant striatal TH depletion after immunohistochemical analyses [53]. On the basis of this previous work, we consider that at the onset of significant motor symptoms, the monkeys in the current protocol have received a significant lesion to the nigrostriatal dopamine system. However, we chose not to sacrifice these highly trained and implanted animals at this moment of the study, and so we are unable to provide histological confirmation of this assertion.

Nevertheless, we acquired PET scans during our protocol, again using the ligand [^11^C]PE2I, to demonstrate that there was modulation of the DA system as previously observed. S4 Fig confirms that across the scans carried out, the MPTP lesion modulates the DA system (repeated measures ANOVA, main effect of scan, Monkey R: F_(4,50)_ = 25.36, *p* < 0.0001; Monkey S: F_(8,94)_ = 17.91, *p* < 0.0001). Importantly, as for the motor symptoms, the two monkeys show the same pattern of modulation over the time-course of the lesion. Specifically, DAT binding is increased above baseline levels at the start of the lesion and then returns to or drops below baseline levels at full dose. This pattern replicates our previous result [53]. Vezoli et al. posited this early increase as a potential compensatory response to DA cell death. Under this interpretation, DA cell death and loss of dopaminergic transmission will be well advanced by the time DAT binding begins to reduce below baseline levels, as they appear to do at the end of the protocol. We can, however, draw only limited conclusions from the PET data set with respect to direct PFC and MCC physiology, due to the low levels of DAT in those regions [55,56]. Binding of DAT in lateral prefrontal cortex was indeed negligible, but S4 Fig shows binding potential of the anterior cingulate region of interest (ROI) (derived from [57]), which includes the region we refer to as MCC [11], as well as for caudate and putamen ROIs. Binding potential in this cingulate ROI is much lower than the striatum, but as in our previous study, DAT binding in cingulate was the highest across all cortical regions, and there is support from immunohistochemical localisation for DAT in this region [56]. We provide these data as indicative. Further studies will require alternative approaches to provide more direct indications of the impact of MPTP on prefrontal dopamine.

We followed the evolution of behavioural and neurophysiological measures throughout the protocol. Figures presented, such as Fig 3C, show the measure in BL (boxplots on left), and then the evolution of the measure with MPTP (lines). The change relative to the BL period is presented as significance bars on the figures. We also tested, at each time-point in the MPTP period, whether the effects reported for the BL period were still significant in and of themselves. These tests are not shown in the figures but are described below.

We considered the RTs to look for early motor changes during the motivated cognitive task. RTs showed no significant slowing despite the dopaminergic lesion (Fig 3C). In fact, RTs showed some sessions with significant speeding in both monkeys (colored bars on Fig 3C, non-parametric comparison with BL using bootstrap, *p* < 0.01 for both monkeys). The reflection of cognitive control in the RTs was significant for Monkey S throughout (ANOVA corrected for multiple comparisons, *p* < 0.01 throughout) but was lost at the onset of MPTP in Monkey R, yet later recovered. Hence, despite the lesion, monkeys maintained similar RT in the task.

### FRPs in MPTP Period

**Fig 4:**
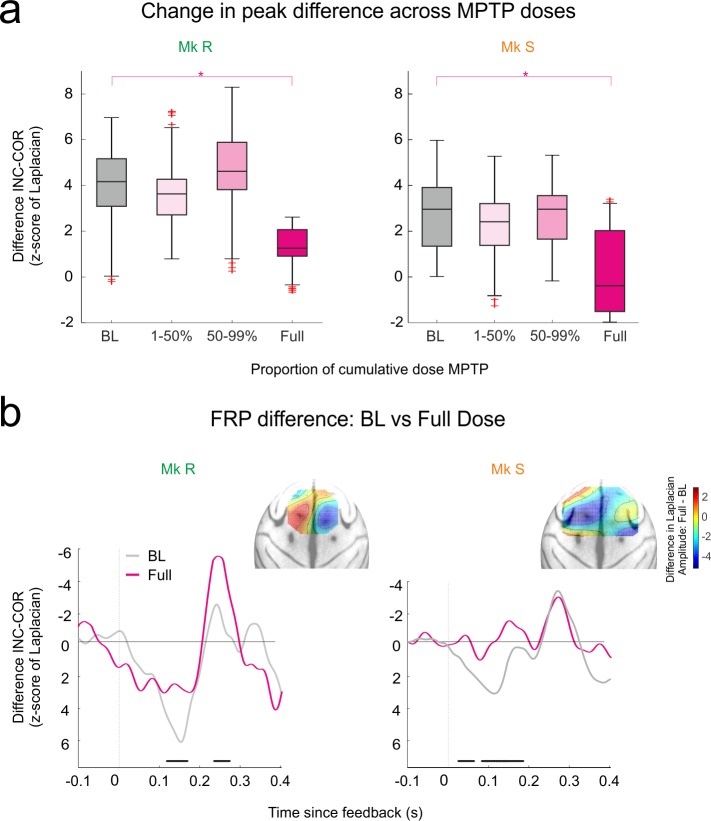
FRPs in MPTP period. MPTP full dose diminishes the capacity of FRPs to distinguish feedback. **A.** Evolution of the difference peak (INC-COR) of FRPs during the MPTP period. Significant difference emerges at full dose only in both cases. **B**. Comparison of the FRP difference wave (INC-COR) between BL and Full Dose. Black bars: permutation test *p* < 0.01 between control and full dose. Insets show the anatomical distribution of the difference in the FRP difference wave (full dose—BL), with projection using the surface Laplacian. The main region of change (reduction of difference amplitude in blue) matches the location of the peak differences in BL (see Fig 2B).

Outcome-related potentials are modulated in some studies in PD, and so we anticipated modulation of the FRPs as a result of our dopamine lesion. At onset and for much of the lesion, FRP difference was maintained (Fig 4A). But at full-dose MPTP, at the end of the protocol, the early peak difference FRP was significantly attenuated in both monkeys (Fig 4A, permutation test between BL and MPTP full dose, *p* < 0.01). Furthermore, there was in fact no longer a significant difference between the correct and incorrect FRP for Monkey S (Fig 4B, permutation test between INC and COR, no significant change from distribution of permutations), although Monkey R did maintain a marginally significant difference (same permutation test, *p* < 0.05). As for the BL period, we performed the same analysis restricted to the SEA phase, to further address whether FRP change is driven by changes in coding the valence of feedback or changes in coding the expectancies. S3C Fig shows that, as in Fig 4A, there is significant attenuation of the INC-CO1 difference at full dose compared to BL for Monkey R. This effect does not reach significance for Monkey S, although the difference between the INC and CO1 is not significant at full dose for this monkey (permutation test between INC and CO1, no significant change from distribution of permutations), with a high variance as can be seen in S3C Fig. It therefore appears that loss of feedback valence coding, rather than feedback expectancy coding, is driving the observed effect. As noted above, however, a design that explicitly equalizes the feedback probabilities would provide a definitive answer.

Analysis of the peak latencies of this difference wave was inconclusive and noisy, and Fig 4B shows clearly why this is the case; after the full dose, a peak difference is no longer truly observed. This attenuation is greatest in the anatomical locations of the original peak (Fig 4B inset, change in peak difference from BL to full dose). This loss of sensitivity to feedback valence in the FRPs might therefore predict impaired performance, if it is the case that performance monitoring signals provide information necessary to adapt cognitive control and efficient choice.

### Cognition and Beta in MPTP Period

**Fig 5:**
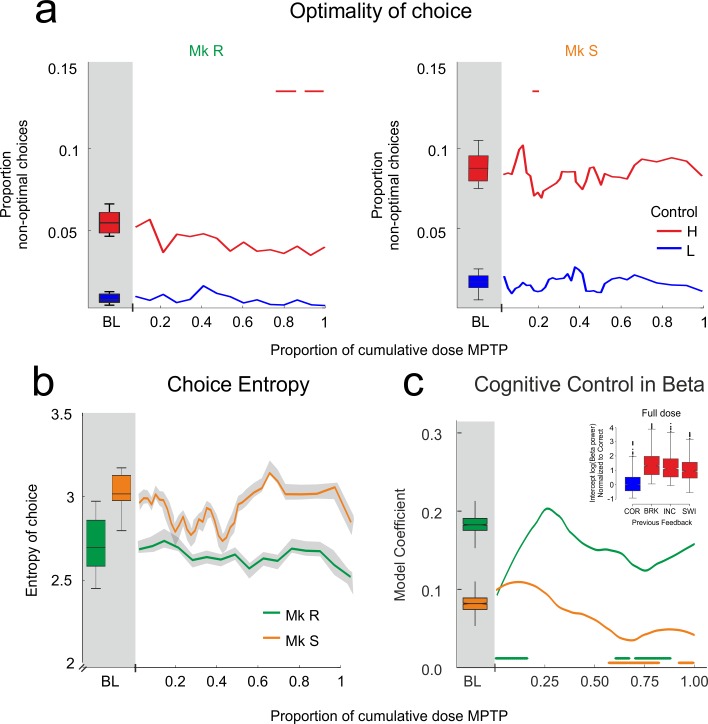
Cognition and beta in MPTP period. MPTP lesion does not impair cognitive control. Cognitive control remains significantly represented in the beta power of the delay. **A**. Change in choice optimality over MPTP treatment. Monkeys remain near optimal throughout treatment. Change with respect to BL only occurs when non-optimal choices reduce, meaning performance improved. **B**. Shannon entropy of the first two choices of the search phase during the MPTP period. The search strategy remains unaffected by MPTP treatment, never varying significantly from BL. **C**. Model-derived coefficient for the cognitive control factor (response to previous feedback) during MPTP period. The solid lines indicate that the factor makes a significant contribution to the model at every point in the MPTP period, so the coefficient remains positive and significant throughout, whilst the colored bars indicate where the coefficient changes with respect to BL. This confirms that cognitive control continues to be represented by beta power throughout the lesion, although the coefficient weakens towards the end of the protocol. Inset: As for Fig 2D, but fitted on data from the full dose—beta power represents cognitive control in the same manner after MPTP treatment as it did before. Colored bars: comparison to BL bootstrap, *p* < 0.01.

Contrary to this prediction, cognitive performance on the PST did not worsen during the lesion. Fig 5A shows that at no point, for neither monkey, and for neither level of cognitive control, did choice become less optimal than in the BL period. More optimal choice on low-control trials was also maintained (ANOVA corrected for multiple comparisons: *p* < 0.01 throughout for both monkeys). The only significant effect was a slight but significant reduction of the proportion of non-optimal choices in high control, meaning improved cognitive performance, particularly in Monkey R (colored bars on Fig 4A, non-parametric comparison with BL using bootstrap, *p* < 0.01). We further tested whether there was an acute effect of MPTP injections that was subsequently compensated for after a few days of recovery. The number of days since the last injection had no significant effect on the proportion of non-optimal responses, despite a trend in Monkey S (Monkey R: F_(1,35959)_ = 0.073, *p* = 0.787; Monkey S: F_(1,40231)_ = 3.73, *p* = 0.054). Finally, monkeys maintained the same level of optimal problem transitions, showing that they continued to take into account the STC (Fig 3B, non-parametric comparison with BL using bootstrap, *p* > 0.1 throughout).

The lack of impairment on the task was somewhat surprising, given that evidence from the literature shows the early emergence of cognitive symptoms in monkeys treated with MPTP [50,58–60] and in cognitively less complex tasks than PST, albeit with higher frequency injections. We discuss this discrepancy below. We further tested whether monkeys’ strategy of initial target choice remained the same, by calculating the Shannon entropy of their first two choices within the SEA phase—that is, how consistent their initial choices were. Notably, this criterion is independent of optimality—initial choice strategy could change, but if incorrect choices were never repeated, search could remain optimal. Throughout treatment, this quantity was maintained (Fig 5B, no change from BL bootstrap, *p* > 0.1).

We next investigated whether the measures of cognitive control reflected in the beta oscillations would be affected as the FRPs were. Cognitive control significantly modulated beta power throughout the MPTP period in both monkeys (statistical model selected on BL and applied throughout the MPTP period using Wald conditioned F tests corrected for multiple comparisons; see Materials and Methods: Monkey R _numerator df = 2, denominator df > 1955_, *p* < 0.0001 throughout; Monkey S _numerator df = 2, denominator df > 2061_, *p* < 0.05 throughout). It is important to stress, therefore, that in both monkeys the reflection of cognitive control in beta power is strongly significant throughout. The dynamic of the coefficient is presented in Fig 5C (solid lines). The significant effect does weaken with respect to BL levels when approaching full dose; but, critically, at full dose when the FRPs are significantly attenuated, both monkeys maintained a significant positive coefficient, Monkey R showing an effect as strong as in BL. We further confirmed that cognitive control did indeed contribute to explaining beta power even after the full dose, by repeating the model selection procedure on full dose data (Fig 5C inset, Wald conditioned F test: Monkey R: F_(2,5395)_ = 83, *p* < 0.0001; Monkey S: F_(2,4211)_ = 3.55, *p* = 0.029, to be compared with Fig 2D). Indeed, the model selection procedure on full dose revealed all of the same factors to be significant as in BL. As such, beta power continued to reflect cognitive control throughout, and related performance was maintained.

**Fig 6:**
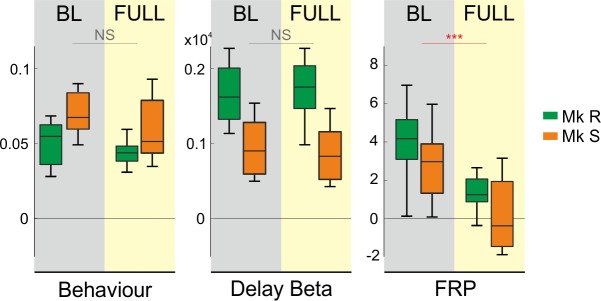
Full dose. Summary of effects on performance, beta power, and FRPs at full dose of MPTP. For both monkeys, cognitive control remains strongly represented in behavioural and delay beta oscillatory measures, in BL, and after full-dose MPTP. By contrast, FRPs show a significantly diminished or absent cognitive control effect at full dose. In each case, the difference measure is shown, and across the measures a zero difference is aligned, to provide an illustrative comparison. Behaviour: difference between non-optimal choice proportion for high- and low-control. Beta: difference in raw power between high- and low-control trials. FRP: peak z-scored Laplacian difference potential between INC and COR trials.

There is, therefore, a striking dissociation pattern in these data, in particular at the full dose of MPTP just prior to motor symptom emergence. The assumed cognitive control loop breaks down; the marker of performance monitoring is attenuated at a moment when the cognitive performance, and the representation of that performance in beta power, is maintained. Fig 6 presents these results at full dose side-by-side for comparison. After MPTP (yellow shading), the behavioural output and beta oscillatory representation of cognitive control are both maintained, whilst the measure of performance monitoring thought to drive these processes was attenuated or lost when compared to the BL period (grey shading).

### Motivation and Beta in MPTP Period

**Fig 7:**
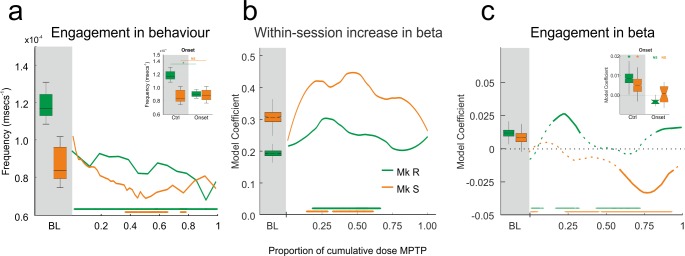
Motivation and beta in MPTP period. Engagement in the task decreases during the lesion, but putative representations of motivation in beta power do not show the same pattern. **A**. Behavioural engagement (frequency with which offered trials were engaged) for the task in BL and MPTP periods. Inset shows the change between BL and the onset of the MPTP period, showing an immediate reduction in engagement of R but not S. Reduced engagement emerges later in the protocol in S. **B**. Model coefficient for within-session increase in delay beta, related to attentional effort. This coefficient remains positive and significant throughout MPTP treatment, as indicated by the solid line. Bars at the base indicate change with respect to baseline. **C**. Model coefficient for engagement factor on delay beta power for BL and MPTP period. In contrast to (B), here the effect in the model ceases to be significant. At these points, we report the coefficient but display it as a dotted line. There is, therefore, only a significant effect where the line is solid. As previously, the bars at the bottom indicate change with respect to BL. Again, inset shows the effect at onset—the influence of engagement on beta is immediately lost at the start of the lesion but is subsequently variable and different between monkeys.

Although cognitive performance was maintained, we did record a behavioural effect of the dopamine lesion—reduced engagement in the task. Engagement is the rate at which the monkeys initiate trials offered to them. Fig 7A shows the level of engagement in BL and then the evolution of this measure over the MPTP period. Monkey R showed reduced engagement compared to BL throughout the MPTP period. Monkey S showed reduced engagement, but later in the MPTP period (colored bars Fig 7A, non-parametric comparison with BL using bootstrap, *p* < 0.01). Indeed, the effect on engagement was present in monkey R from the very onset of the MPTP treatment (Fig 7A inset, same test, *p* < 0.01), demonstrating a rapid effect of the lesion. We conceived the engagement measure as an index of motivation. It must be noted, however, that this interpretation is not unambiguous: a motivated monkey will engage quickly, but a monkey applying high cognitive control might engage more slowly to ensure optimal performance.

There is therefore a behavioural dissociation between cognitive performance and motivation. We sought to understand whether the beta power during MPTP lesion reflected the changing engagement as well as the maintained cognitive control. In the BL period, time within-session (the attentional effort effect) and engagement frequency both significantly contributed to explain beta power (Fig 2). Both factors could arguably be related to motivation, and neural markers have been proposed as a manner of understanding the complicated relationship between motivation and cognitive control [4].

The remainder of Fig 7 shows the dynamic of these influences on beta power throughout the MPTP period. Within-session time, the factor linked to attentional effort, continued to significantly influence beta power throughout the MPTP period (Fig 7B, Wald conditioned F tests as above: Monkey R: _numerator df = 2, denominator df > 1955_, *p* < 0.015 throughout; Monkey S _numerator df = 2, denominator df > 2061_, *p* < 0.0001 throughout). So attentional effort modulates beta power despite the dopamine lesion. Fig 7B shows a strengthening of this effect in the middle of the protocol, potentially signaling a compensatory increase in attentional effort to maintain the good performance as the lesion continues.

In contrast to this, the trial-by-trial engagement frequency immediately ceased to influence beta power at the onset of the MPTP period (Fig 7C inset, Wald conditioned F test: Monkey R: F_(1,6216)_ = 0.53, *p* = 0.46; Monkey S: F_(1,5340)_ = 0.26, *p* = 0.61), potentially reflecting the early behavioural effect, although the behavioural effect at onset is limited to one monkey. But this loss of effect was not permanent, and indeed Fig 7C shows that the influence of engagement on the beta power varied non-linearly throughout the protocol, reflecting neither the behavioural engagement nor the maintained within-session effect.

## Discussion

Our data reveal a nonlinear chain of neurophysiological, cognitive, and motor changes that emerge in response to a progressive dopamine lesion. Monkeys performed near optimally a test of cognitive control throughout slow low-dose MPTP treatment, before the development of significant motor symptoms, and in the context of compensatory alterations in DAT levels. During this time, prefrontal beta oscillatory power continued to represent the cognitive control demands of the task trial by trial. However, the engagement of the monkeys in the task and the representation in beta power of that engagement were modified during the lesion. The performance monitoring FRP signals thought to inform the use of cognitive control were maintained for much of the protocol. But at full dose, just prior to the emergence of clinically relevant motor symptoms, monkeys showed attenuated differences between the evoked potentials to correct and incorrect feedback, even though they were still performing the task well.

Our results dissociate markers of performance monitoring, motivation, and cognitive control, posing important questions for our understanding of the systems involved. It is already established that good performance on this task cannot be explained by simple reinforcement learning alone [13]. So whilst performance monitoring is important to the task, it also requires an implementation of cognitive control that has neurophysiological correlates in MCC and prefrontal cortex [12,13,23]. The dopamine lesion appears to selectively impact the potential biomarker of performance monitoring—the FRP. However, change in the FRP fails to correlate with cognitive change. When a biomarker behaves in such a manner, only two interpretations are possible. First: a compensatory process is replacing the putative function of the biomarker. Or second: the biomarker is not in fact performing the function it is assumed to perform. We address these two possibilities in turn.

### Compensatory Processes

The proposal that dopamine-influenced MCC performance monitoring signals do drive subsequent adaptation of behaviour [10,61,62] remains a mainstay of the current literature. Perturbation or lesion of the MCC leads to behavioural impairment, at least over choice sequences [63–65], whilst neurophysiological signals in MCC clearly show trial-by-trial adjustment on the current task [12,13,66]. Reinforcement-learning theory of medial frontal performance monitoring signals hypothesizes a link with dopamine [10], and these signals are modified in some cases of diagnosed PD [40,43,44]. We found attenuated FRPs after dopamine lesion. Yet despite this change, behaviour remained near optimal, and the beta power continued to represent trial-by-trial control levels and adjustments. A compensatory change of the performance monitoring system may therefore occur as the dopamine system is modulated, maintaining cognitive control and performance. Compensation might occur through network [67] and/or neurochemical [68] changes.

Ascribing a behavioural non-impairment after lesion to compensation can become an uninformative catch-all interpretation. The power of combining a lesion approach with neurophysiology is that we can provide evidence for and a potential source of that reorganisation. Here we show that the beta oscillatory cognitive control signal is maintained (albeit weakened slightly at the end of the protocol), so the focus of any compensation should be the source of FRP, putatively MCC. It is therefore tempting to conclude that the MCC has reorganized in the face of changing dopamine input, in order to continue to provide necessary information to the control system, even though the FRP signal itself is lost. Our PET data provide tentative support for this interpretation, in that they indicate an increase in binding to DAT in cingulate regions in the early phase of the lesion, a phenomenon also observed in striatum and ascribed a compensatory role in our previous work [53]. Binding to DAT is then decreased at full dose, when the FRP is modulated. A weakness with this interpretation, however, is that there remains a lack of conclusive evidence that (1) the FRP has its source in MCC, and (2) that the FRP is a direct product of dopaminergic prediction error signals in MCC. Answers to these questions require direct inactivation or dopaminergic modulation locally within MCC.

### An Alternative Role for FRP

The alternative to this compensatory hypothesis is to conclude that the FRP is not a signal necessary for trial-to-trial adaptations of control. The alternative proposition would be that the actual trial-by-trial adaptations are mediated by the striatum [69], whilst the MCC and the dopaminergic input it receives would be the source of a motivational control signal driving the selection of extended behaviour sets (options) [15,17,69,70]. An increasing body of work questions the direct trial-to-trial influence of the FRP on behaviour. For example, a simple behavioural modulation such as the provision of task contingencies is sufficient to dissociate changes in the FRN from behaviour in human subjects [16].

Under this hypothesis, the changing FRP that we observe is indeed a marker of the dopamine lesion but should not be expected to lead to immediate cognitive optimality deficit, but rather an impairment in behavioural set selection, which might manifest itself in reduced motivation. This interpretation is consistent with our previous work showing modulation of the FRP in this task following systemic injection of the dopamine antagonist haloperidol, in the context of maintained cognitive performance but reduced motivation for the task [8]. In the current study, the significant reduction in motivation in both animals (Fig 7A) further supports this argument. More complicated to interpret is the relationship between motivation measures and beta power. Trial engagement effect on power was immediately abolished (Fig 7C inset) but then followed a dynamic that did not match the continued reduced behavioural engagement in both monkeys. Such a pattern might itself be a signal of compensatory processes and represents a target of future study. In contrast to this, the within-session increase in beta power remained a strongly significant factor throughout (Fig 7B). Indeed, this effect even strengthened in both monkeys with respect to BL in the middle part of the protocol. We discuss the latter effect further below. Lowered behavioural engagement is reminiscent of apathetic symptoms in PD. Our frontal markers only partially reflect this effect, and separation of motivational functions within these signals may require careful computational analysis [71], a topic for future work. Nevertheless, our results add to a literature questioning a simple mechanistic link between dopamine depletion, neurophysiology, and apathy in PD [72].

A second element that may dissociate FRP from trial-by-trial performance is overtraining. Premorbid practice is known to have a protective effect on cognitive tasks [73]. As in the majority of monkey neurophysiology protocols, our animals were extensively trained on the cognitive task. It is important not to forget, however, that monkeys were already well trained in the BL period when there was a large FRP. Moreover, even a well-trained monkey is obliged to take feedback on each trial into account in order to display the near-optimal performance we report in Fig 5A. Automatic responding cannot lead to such performance, and this questions whether truly habitual performance is possible in this task. Nevertheless, it has already been suggested that over-training might diminish the need for differential feedback signals and the related dopaminergic signals in trial and error adaptation [74,75]. Thus, it may be possible that dopamine prediction error-driven modulation of the feedback response is necessary to learn the significance of feedback, but not to perform the task once this is learned. Longitudinal recordings throughout the long training protocol for such a task are necessary to reveal any such effect. The difference signal we report in BL would therefore be a no-longer-necessary residue of this process, hence the lack of behavioural effect of an attenuated difference signal.

### Beta Power and Control Implementation

In contrast to the effect of MPTP on the FRP, modulation of beta power with control implementation remained significant throughout, although reduced compared to BL at the end of the protocol. The relationship between this beta signal and established dopamine-sensitive neural correlates of cognitive control and working memory remains an open question. Cells in PFC that respond to this task show delay activity, as in working memory (delayed response) tasks, and this delay activity is modified by control demands in different phases of the task [18]. PFC delay activity relies on DA [45] in multiple ways, in that D1 receptor modulation impacts tonic delay activity and related behaviour [32,34,46], whilst D2 receptor modulation impacts phasic activity [33]. These separate roles are hypothesized to relate to differing dopamine-induced states that drive maintenance and robust representations through D1 [76] and transitions and flexibility through D2 [25].

It is unclear how the beta oscillations that we observe relate to this established single-unit activity in the prefrontal cortex. Delay beta power occurs at the same delay period as the single unit activity in [18], and both phenomena are more pronounced when control demands are high (Fig 5B of that study, Fig 2D here). We can therefore speculate that they reflect similar processes. Under this hypothesis, within-session increase in power may relate to an increased D1 response impacting single unit and beta delay activity that modulate maintenance in the face of distractors and fatigue, thereby leading to an attentional effort effect. To our knowledge, within-session analysis of single-unit delay activity along similar lines is absent from the literature, but it will be the target of future study. On this basis, the increase in within-session effect under early MPTP lesion reported in Fig 7B might represent an augmentation of this effect, providing compensation for the lesion. Two observations from our study support this assertion. First, as the within-session effect in Fig 7B returns to baseline levels, at around 0.65 of full dose, so the cognitive control effect in Fig 5C begins to weaken. Second, the increase in within-session effect coincides with the increased DAT binding in the striatum (and putatively cingulate cortex) that we have associated with compensatory responses to the MPTP lesion [53]. Meanwhile, the lack of impairment on the task in terms of cognitive optimality suggests we are having little impact on D2 mediated processes within this lesion, in that D2 has been linked to updating processes necessary for adapting to changing problems [25]. Again, these speculative interpretations are the subjects of future study, but it is increasingly clear that careful dissection of motivational, motor, and cognitive deficits is an important future route for DA and PD research [71].

### Lesion Pattern

In PD and some progressive MPTP protocols, the lesion progresses dorsally to ventrally within the striatum [52,77]. This pattern of degeneration suggests greater impact on motor than cognitive functions, and has been linked to later development of cognitive symptoms and the ambivalent effects of dopaminergic medication on cognition [78]. Our behavioural results are consistent with this account in that we see no cognitive impairment before motor symptoms appear. They are inconsistent with previously reported premotor cognitive impairments [50,58–60]. But we record frontal neurophysiological changes early in the lesion and, in particular, FRP modulations prior to significant motor or cognitive symptoms. If these changes precede alterations of motor neurophysiology, they are incompatible with the dorsal-ventral account. We are unable to provide histology at each phase of the protocol to investigate lesion progress in terms of both pattern within striatum and impact on PFC and MCC. This would require sacrifice of a large number of animals. The measure that we recorded using PET (DAT binding using radiotracer [^11^C]PE2I) provided good indication of striatal DAT levels, but in cortex, DAT levels are low and only cingulate DAT binding was significant. We have confirmed that MPTP at this dose induces a putative compensatory increase in DAT, followed by a global loss of DAT binding once motor symptoms becomes significant [53].

Here we have revealed the dynamic of a multidirectional relationship between dopamine, motivation, and cognitive control over longitudinal dopamine depletion. Future work must focus on the extent to which these dynamics can be ascribed to the dopamine lesion itself or the compensatory processes combatting it.

## Materials and Methods

### Ethics Statement

Ethical permission was provided by “Comité d’Éthique Lyonnais pour les Neurosciences Expérimentales,” CELYNE, C2EA #42, ref: C2EA42-11-11-0402-004. This permission endorsed our MPTP safety protocol, drawn from published NIH guidelines for all elements of MPTP use and housing of treated animals. Monkey housing and care was in accordance with European Community Council Directive (2010) and the Weatherall report, "The use of non-human primates in research." Laboratory authorization was provided by the "Préfet de la Région Rhône-Alpes" and the "Directeur départemental de la protection des populations" under Permit Number: #A690290402. This article has been written to comply with the ARRIVE guidelines for reporting animal research, and an ARRIVE checklist forms part of the supporting information.

### Subjects and Materials

Two rhesus monkeys (*Macaca mulatta*)—Monkey R, a 17-y-old female weighing 7 kg, and Monkey S, a 16-year-old male weighing 8.5 kg—served as subjects. Monkeys were trained in a recording box, seated in a primate chair (Crist Instrument Co., Hagerstown, MD, USA) and in front of a tangent touch-screen monitor (Microtouch System, Methuen, MA, USA). An open window in front of the chair allowed them to use their preferred hand (monkey R, left-handed; monkey S, right-handed). All elements of the task were controlled and recorded on a PC running the CORTEX software (NIMH, Bethesda, MD, USA). Eye movements were monitored using an Iscan infrared system (Iscan Inc., Woburn, MA, USA). Electrophysiological data were recorded using an Alpha-Omega multichannel system (Alpha Omega Engineering, Israel). We reported details of tasks and implantation of these monkeys in [23], but reproduce important elements below.

### Behavioural Tasks

#### Problem solving task with 4 targets (PST4)

The PST was developed from classical working memory paradigms in order to add a level of control requirement onto the simple delayed-response paradigm. As a team, we have demonstrated the detailed neural correlates of both the feedback processing and behavioural adaptation elements of the task in both monkey and human subjects [12,13,18,23,66,79]. Specifically, the task induces delay activity in PFC comparable to that classically observed in working memory [18], induces responses to feedback that are modulated by control levels [12] and sensitive to dopamine [8], and explicitly requires control in that performance cannot be explained by simple reinforcement learning [13].

Monkeys sought, by trial and error, the correct target from a choice of four (Fig 1A) during the search phase (SEA). Once they had found the rewarded target, they entered the repetition phase (REP). During REP, the rule remained the same, and monkeys could repeat the rewarded choice for three additional rewards. Successful SEA, discovery of a rewarded stimulus, and REP of that response three times is hereafter termed a problem. Having completed a problem, monkeys saw a “signal-to-change” (STC) on the screen. The STC announced the re-pseudo-randomization of the rewarded location, and therefore instructed monkeys to begin SEA again.

Each trial, regardless of phase, followed an identical format. Monkeys initiated the trial by touching a grey triangle (the “lever”), then fixated a fixation point (FP) for a delay of 1,400 ms. This delay epoch was important for subsequent analyses. Next, four grey target circles were displayed on the upper side of a circular axis (Fig 1A). At the onset of targets (ON signal), monkeys made a saccade towards their selected target and fixated it (random delay 400, 600 or 800 ms). All targets now turned from grey to white, providing the GO signal and prompting the monkeys to touch the target they had already chosen by fixation. After a further random delay of 600 to 1,200 ms (steps of 200 ms), a visual feedback stimulus was shown to the monkey for 800 ms. Feedback was horizontal (correct) or vertical (incorrect) rectangles, in the same location and of the same grey as the circular targets. If the choice was incorrect, there was negative visual feedback and no reward, whereas correct responses were rewarded with positive feedback followed by a 1 or 1.8 ml pulse of fruit juice. To maintain stable performance and increase motivation, monkeys received a large reward bonus (20–30 ml of fruit juice) if they completed a fixed number of problems (*n* = 110 & 60 problems for monkey R and S respectively). Poor execution of a trial was recorded when monkeys made a break in fixation or an inaccurate touch. These trials were immediately interrupted with a signal informing the monkey that this was a break trial (BRK).

#### PST2

The PST2 task was identical to PST4, with the sole exception that there were only two stimuli presented throughout, pseudo-randomly selected from the four possible stimuli used in PST4. SEA in PST2 is therefore easier for the monkeys. REP was unchanged, requiring three correct responses. PST2 and PST4 problems were presented pseudo-randomly in the same sessions.

### Behavioural Analysis

#### Task performance

Cognitive performance on PST was measured as a proportion of non-optimal choices. When the monkey is searching, the commission of errors is to be expected as an optimal part of evidence accumulation, but repetition of those errors is not. Hence, in SEA, non-optimal choice was the proportion of trials on which the monkey repeated an incorrect choice already made during that SEA. During REP, the monkey has found the correct response and must simply repeat it three times. Hence, the non-optimal measure is the proportion of incorrect choices during REP. We further tested whether monkeys used the STC to re-initialize their search at the start of a new problem. This requires the monkey to immediately change chosen target compared to the previous REP, because each new problem has a new pseudo-randomly assigned correct target, and the chance of the newly assigned correct response being the same as the previous one was very low. Note that monkeys must therefore use STC to override correct feedback, as the last trial of REP is by definition correct.

As a final measure of cognitive strategy, we calculated the Shannon entropy of monkeys’ first two choices within the SEA phase, applying the calculation:

$$H=\sum_{i}p_{i}\;{log}_{2}(1/p_{i})$$

where H is the calculated entropy and p_i_ the probability of each possible transition between first and second choices of target. This therefore provided a measure of how consistent their initial choices were in terms of the individual stimuli chosen (i.e., did the monkey prefer to start searching on stimulus 1, then stimulus 2, etc.). This measure was of interest in the context of potential cognitive changes with the dopamine lesion, and in particular because it is independent of optimality—the monkey could acquire or lose an initial choice preference, but if incorrect choices were never repeated, search could remain optimal.

Execution of the task was reflected in response time (RT), the time between the appearance of white targets (GO signal) and the monkeys’ touch of the chosen target. Across treatment response time could potentially act as a sign of motor impairment. This response epoch can alternatively be divided into reaction time (the time until the monkey releases the lever in order to start the response) and the subsequent movement time. We prefer here to present response times that combine these measures, as there is no guarantee as to how the monkey will choose to apply control within this epoch; for example, control-related slowing might be applied just before the release (long reaction time) or during the movement itself (long movement time). Use of response times captures both possibilities. Nevertheless, S2 Fig confirms that we obtain effects of cognitive control on pure reaction time measures just as we do on the response times.

The ongoing “engagement” of the monkeys in the task was used as a proxy measure for their motivation. We calculated an engagement rate from the time between the end of the previous trial and the first touch of the monkey on the lever at the start of the following trial. Monkeys were free to touch the lever when it was on the screen at any moment after the ITI. The assumption is that monkeys will engage more quickly when more motivated. In order to render this measure orthogonal to cognitive performance, we normalized for each trial outcome type. This also accounted for slight timing differences for the end of the trial between correct trials (which end in juice reward) and incorrect trials (which end after negative feedback). A further parameter analyzed with relation to timing was simply the time of each trial relative to the start of the session. This time was then normalized to the mean of all of these timings in order to center the measure. This permitted us to study measures that changed over time within-session.

#### Application to neurophysiology

Cognitive control is a computational process rather than a psychological function, and so to study it we induce variable use of cognitive control across a common trial structure, and then show how neurophysiological processes are modulated with cognitive control demands. “High-control” trials are those in which the monkey must adapt behaviour following the outcome of the previous trial; this can be after an incorrect choice (INC), a switch due to the STC (SWI), or after a break in fixation or touch (BRK). These three classes of high-control trials contrast the trials that follow a correct response (COR), when the monkey simply has to repeat the response, making this a “low-control” trial. We have a well-established record of using this task to detect cognitive control processes in neurophysiological data [12,13,18,23,79].

### Surgical Procedures

Each monkey received an implant consisting of a head-post and a grid of transcranial electrodes for ECoG recordings. We performed the implantation surgery in aseptic conditions. Monkeys received full anaesthetic along with appropriate antibiotic and analgesic treatments during and after the surgery, along with extensive monitoring. Details of the doses administered and further surgical detail can be found in [23].

We had previously acquired structural MRI images of each monkey, and we used these scans to guide the implantation location of the electrodes and to ensure consistent depth of insertion across electrodes. The MRI images provided us with stereotaxic coordinates at which we drilled individual holes through the skull. We then screwed stainless steel surgical screws (Synthes) into each hole, such that at each site the end contact point of the screw rested on the dura mater, acting as an ECoG electrode. The grid of electrodes was then soldered to a connector constructed in-house, to permit the daily recordings. The electrodes, connectors, and head-post (Crist Instrument Company, USA) were anchored together with dental acrylic.

Schematics of the electrode grids can be seen in S1C Fig. Monkey R received a 5-mm-spaced grid of 14 electrodes over prefrontal cortex. This monkey then received a further eight electrodes over sensorimotor cortex and around the central sulcus in a second operation (S1C Fig, left panel). Monkey S received a larger 7-mm-spaced grid of 31 electrodes in a single operation. Again, the implant covered the prefrontal and sensorimotor cortex (S1C Fig, right panel). Finally, we implanted a single reference electrode in each monkey, in the form of an additional screw inserted into the think bone of the brow, on the midline and well anterior to the most anterior prefrontal electrodes.

### MPTP Intoxication

The BL period provided a baseline for all measures and allowed habituation to sham injections of MPTP. Sham injections were given at the end of each week, in exactly the same safe conditions as subsequent MPTP injections. Sham injections were 0.5 ml sterile water IM. The BL was, respectively, 46 and 55 d for monkeys R and S. The MPTP protocol was then induced.

We employed a chronic low-dose protocol with injections of MPTP. Treatment consisted of i.m. injections of MPTP-HCl (Sigma M0896) diluted in sterile water at 0.2 mg/kg. The aim of the protocol was to induce a dopaminergic system lesion modeling the slow rate envisioned in the premotor period of PD [51], and to permit concurrent electrophysiological recordings. The protocol used a dosage of 0.2mg/kg, well established in progressive protocols of MPTP [49,50], but delivered this dose and therefore attained symptoms much more slowly.

MPTP injections were performed a maximum of once per week, at the end of a week of recordings, in the home cage, without prior sedation. Monkeys remained in the home cage for 72 h after each injection, with ad lib access to water and food, and were regularly monitored. There was both a safety aspect (allowing time for the removal of MPP+ from the excreta) and an experimental aspect (avoiding any acute injection effects) to this procedure. After this time, the monkey would resume work and recordings until the next injection. The typical schedule in this protocol was, therefore, as follows: work and recordings from Monday to Friday, MPTP injection Friday afternoon, recovery during the weekend, and restart of work the following Monday.

We used the Parkinsonian Monkey Rating Scale, adapted from the UPDRS and compatible with rating scales used for monkeys [50,53,80,81] to score symptoms and judge the cessation of treatment. At least two authors and a colleague blind to the aims of the experiment scored monkeys through the week, giving weekly average scores. As previously, we used the motor subscale of the PMRS [50]: 0 = complete absence of motor symptoms; 1–5: pre-symptomatic, slight but observable symptoms; and 5 = clinical threshold, adjudged to be the equivalent of diagnosed symptoms in a human. The end of the MPTP period was when monkeys reached a score of 5 or more. In practice, both monkeys ceased to work for the task reliably in the week that their symptoms reached a score of 5, so we considered only data up to and including the dose prior to this. The “full dose” period therefore refers to the sessions in the 3 weeks prior to the week during which monkeys reached a score of 5 (Fig 3A, Monkey R 14 sessions, Monkey S 15 sessions).

To test for acute effects in the week after injection, monkeys had regular “recovery” fortnights, during which the testing protocol continued in identical fashion, but there was no MPTP injection. These recoveries were spaced with decreasing frequency throughout the protocol: the number of weeks of MPTP injections between recovery weeks was as follows: 2, 2, 2, 3, 3, 4, 5. After this, Monkey R completed the protocol, and Monkey S continued with 5 weeks of MPTP between recovery fortnights.

Induction of symptoms up to the clinical motor threshold requires a significantly increased cumulative dose in slower, progressive protocols [50,51]. Our slow protocol is representative, as the cumulative dose required to bring monkeys to 5 on the PMRS was coherent with the previously observed range [50]; Monkey R required a cumulative “full dose” of 3 mg/kg, and Monkey S required 10.6 mg/kg. Importantly, the study brought both animals to symptomatically equivalent states as the functional endpoint of the protocol; both attained a clinical score on PMRS of 5, and both ceased to work on the task at this point. From these functional points of view, therefore, the two cases can be considered comparable, and the pattern of PMRS scoring across the MPTP period up to full dose was highly comparable (Fig 3A). Progression of the lesion is presented as the proportion of the cumulative “full dose,” a proportion of zero referring to the BL period.

### PET Analysis

Detailed descriptions of the PET protocol and analysis methods can be found in full in [53]. We reproduce the essential elements here. We obtained images from PET scans using (E)-N-(3-iodoprop-2-enyl)-2beta-carbomethoxy-3beta-(4′-methylphenyl)-nortropane labelled with carbon 11 ([^11^C]PE2I) throughout the protocol. [^11^C]PE2I has high affinity and selectivity to DAT and is used to index the integrity of the DA pathway [55,82]. We used an ECAT Exact HR+ tomograph (Siemens CTI), in 3D acquisition mode, covering an axial distance of 15.2 cm. The trans-axial resolution of the reconstructed images was about 4.1 mm full-width and half maximum in the centre. Transmission scans were acquired with three rotating ^68^Ge sources.

We anaesthetised monkeys with 15 mg/kg Zoletil (Tiletamine & Zolazepam, Virbac, France) after premedication with 0.1 mg/kg atropine sulphate, and placed them in the scanner in an MRI-compatible stereotaxic frame (Kopf, CA, USA). We injected [^11^C]PE2I as a bolus followed by a saline flush through a cannula in the femoral vein. Radioactivity was measured in a series of 24 sequential time frames of increasing duration (from 30 s to 10 min; total time 70 min).

We used the anatomical-MRI and maximum probability atlas [57] to define 88 ROIs. Anatomical MRI acquisition was performed in a different session and consisted of a 3D anatomical T1-weighted sequence using a 1.5-T Siemens Magnetom scanner (Siemens AG, Erlangen, Germany). The anatomical volume covered the whole brain with 0.6 mm cubic voxels.

The registration and transformation process to allow extraction of regional PET time activity curves (TACs) of the 88 ROIs is described in detail in [53], as is the quantification of regional [^11^C]PE2I non-displaceable binding potentials (BP_ND_). Because this procedure is semi-automated, we repeated it within and between two experimenters independently in order to account for within-measure variance. For each monkey, a PET scan was acquired in the BL period (prior to MPTP) as well as at regular intervals throughout, and once for each monkey during the 3-week “full dose” period. As in the previous study, cortical levels of [^11^C]PE2I-BP_ND_ were much lower than striatal levels, but the distributions of cortical and striatal DAT did overlap (ranksum, *p* > 0.05). These highest cortical DAT binding values were in the cingulate cortex ROI of [57].

Given the importance of ongoing recording of neurophysiology and cognitive performance to the protocol, and the necessity of anaesthetising the animals, PET scanning was limited to a single radioligand and to three to eight weekly intervals. Note that [^11^C]PE2I-BP_ND_ is a specific measure of the presence of the dopamine transporter, and cannot be considered as a direct index of dopamine levels. Note also that there are more PET scans for Monkey S as a result of the longer treatment period necessary to induce threshold symptoms. Future research on this topic will be required to link the neurophysiological and behavioural results here with more detailed consideration of cortical DA function under MPTP lesion.

### Statistical Testing

All statistical testing was performed within-monkey. During MPTP intoxication, we tested two results at each time-point throughout the lesion. First, we tested whether the measure in question had changed with respect to BL. To do this we used a non-parametric approach: we constructed a bootstrap distribution on the BL period data by resampling with replacement 10,000 times. Then we compared each MPTP period value with that bootstrap distribution. This bootstrap comparison is represented on the figures of the MPTP period as the statistical significance bars, as described in the figure legends.

Second, we tested whether the measure in question still showed the same between-condition effect as reported for the BL period. For example, in Fig 3C we test whether the difference in RT between high and low control trials reported for the BL period is maintained at each time-point in the MPTP period. To do this we repeated the test applied in the BL at each time-point and then corrected for the multiple comparisons using a conservative Bonferroni approach. These statistics are presented in the results section. For the model coefficients in Figs 5 and 7, the significant coefficients are shown on a solid line, whereas the non-significant coefficients are still shown for illustration, but on a dotted line (notably in Fig 7).

Individual statistical approaches for the different electrophysiological analyses are described below.

### Electrophysiological Data Processing

Electrodes were referenced to the frontal reference electrode (S1 Fig). The signal was amplified and filtered (1–250 Hz), and digitized at 781.25 Hz. Data analysis was performed with FieldTrip toolbox [83] and in-house Matlab scripts (Matlab, The MathWorks Inc., USA). Movement artifacts were removed by decomposing ECoG recordings with an independent component analysis (ICA) using the logistic infomax algorithm [84].

For analysis of induced oscillations in the delay epoch, we aligned single trial data to the target onset (ON signal), whilst for analysis of evoked responses to feedback we aligned to the onset of the visual feedback. In addition to the evident information being processed just after presentation of feedback, we focused on the delay epoch because this is when the monkey is integrating feedback information from the previous trial with preparation for the upcoming trial. It is therefore likely to be a moment of implementation of cognitive control [23].

All electrophysiological analyses were developed on data from BL and then applied to the data from MPTP treatment.

#### ERP analysis

We filtered the data (high pass = 1 Hz, low pass = 30 Hz) and then removed artifacts by visual inspection of individual traces and thresholding. We then performed a Laplacian transformation on the ECoG signal to increase the spatial resolution, following the procedure of [85]. Any linear trend throughout the feedback epoch was removed and event related averaging was carried out on the Laplacian transformed signals using *ft_timelockanalysis* in FieldTrip. A baseline epoch of the 200 ms prior to feedback onset was used and subtracted from the data using *ft_timelockbaseline*.

In order to generate reliable numbers of trials, data were concatenated across the sessions performed in a given week, in general 5 days of work. Sessions within the same week were always at the same cumulative dose. Sessions with fewer than 20 problems were excluded. For grand average measures and subsequent statistical analysis, weeks at the same dose of MPTP were further averaged using *ft_timelockgrandaverage* to generate FRP control figures in Fig 2 and MPTP figures in Fig 4. It should be noted that our electrode placement and referencing bear no relation to those used in human EEG studies, and so whilst we expect FRP differences between feedback types, we do not expect waveforms that resemble those well-established in human studies. Individual electrodes for display were those showing the greatest numerical effect between incorrect and correct feedback, whilst surface map constructions used *ft_topoplotER* on the Laplacian transformed FRPs. For statistical analyses of the difference in FRPs between conditions we applied the method described by Maris and Oostenveld [86]: non-parametric permutation tests. This well-validated approach allows simple resolution of multiple comparison problems and avoids some common pitfalls of parametric testing. We applied this approach to test both between INC and COR FRPs and to test the difference waves between BL and MPTP. For all permutation tests, we first generated a test t-statistic on the comparison in question from each time bin, keeping only significant clusters of bins [86]. Next, we collected together trials from the two conditions in question (INC versus COR, or BL versus MPTP), randomly partitioned the trials into two subsets, and calculated a t-statistic between the two partitions. We repeated this process 10,000 times to generate a permutation distribution and then compared the test t-statistic of the correctly partitioned data to that of the permutation distribution, accepting as significantly different any test statistic falling outside 99% of the permutation distribution (thereby effectively setting a threshold of *p* < 0.01).

We initially used this approach to analyze INC versus COR differences in BL FRPs (Fig 2A). Next, we applied it to compare MPTP period with BL. We split the MPTP period into three phases according to the progression towards the full dose: 1%–50%, 50%–99%, and 100% of full dose. We took a bin of data at 25 ms on either side of the peak difference from the BL (Fig 2B) and applied the permutation test approach to compare in turn each of the three MPTP bins with the BL bin (Fig 4A).

#### TF analysis and mixed-effects models

We used convolution with complex Gaussian Morlet’s wavelets with a ratio f/δf of 12 using the command *ft_freqanalysis* to extract trial-by-trial power. The continuous ECoG data were epoched from -2,500 to 2,000 ms (by steps of 10 ms), and we computed the power of each frequency ranging from 4 to 40 Hz in 0.5 Hz steps. We inspected power spectrum density representations independent of influence of the task in order to extract frequencies of interest in the oscillatory activity. As in a previous study [23], we extracted two separate peaks of beta oscillatory power in each monkey, albeit at different frequencies in the two (beta1: 15–18 Hz and beta2: 20–24 Hz for Monkey R; beta1: 10–18 Hz and beta2: 24–32 Hz for Monkey S). When these were averaged trial by trial over the delay epoch (-1,200 to -200 ms before the ON signal) and compared in the SEA and REP phases, we again saw significant differences in power only in the beta2 range. As a result, we focused our analyses on this well-established signal, and henceforth beta refers to beta2 power.

We fitted trial-by-trial beta power measures with linear mixed-effects models [48,87]. Such models allow us to analyze hierarchically organized data and to explicitly model variance inherent to repeated measure designs. The detailed principles and further methods of this approach are described in [23]. All statistical procedures were performed using R (R Development Core Team 2008, R foundation for Statistical computing) with packages nlme and MASS.

To model trial-by-trial beta oscillatory power in the delay from the BL, we used the following factors (and levels): Session, Previous trial feedback factor referred to as PFB (Break, Incorrect, Correct, Switch), Task (PST2 / PST4), and the covariates “time” (time of target onset from the start of session, or within-session time), Response Time (RT), and engagement frequency (Eng). Here, the Switch case refers to trials after an STC. We have previously established an effect of within-session time on beta power in this task [23] and linked it to the concept of attentional effort, because the within-session increase is reset by voluntary pauses in work and therefore reflects a more complex variable than simple time-on-task. We therefore refer to the influence of this factor as the effect of attentional effort. We log transformed the dependent variable Beta (trial-by-trial beta power measured in the time window of interest), following analysis with a Box-Cox power transformation. Hence, log(Beta) was used as a dependent variable.

Models were first selected using BL period data acquired on one test electrode in each monkey, and then applied to all electrodes. There is a discontinuity in within-session progression of beta power when the monkey chooses to take a break in work, something monkeys are free to do in PST [23]. We therefore limited current analyses to the “main” long bout of work in a given session, requiring a minimum of 50 consecutive trials, and ceasing when the monkey made a pause of more than 2 minutes. Any session without a qualifying bout of work was excluded.

We constructed a full model with all possible covariates and factors, and as a first stage of model selection compared models with and without specific random effect terms. Random within-session slopes and daily intercepts for sessions were retained (L.ratio test, *p* < 0.0001 in both monkeys), and these random effects were included in all subsequent models. We later performed model selection on data after MPTP full dose (see below) and confirmed that these random effects terms improved the model after, as well as before, the lesion.

We then evaluated the contribution of fixed effects by repeatedly testing the effect of dropping the highest-order interaction fixed-effect term on the fit [87]. The selected model was fitted on all electrodes by incorporating the factor electrode as an overall interaction term. We had previously validated this approach [23], and again confirmed it on the current data. The final selected model for beta power was as follows:

$$log(Beta)={(\beta }_{0}+b_{0,S})+{(\beta }_{t}+b_{1,S})time+\beta_{PFB}*PFB+\beta_{Eng}*ENG+\varepsilon$$

where the βi are fixed effect and the bj random effect coefficients, each of the latter assumed to be distributed as *N*(0; σ_j_^2^). The subscripts are coded as S-session, t-time, PFB-Previous-feedback, and Eng-Engagement. PFB is a behavioural factor, whilst time and Eng are covariates.

We validated models by checking that normalized residuals plotted against fitted values showed no systematic trends, and confirming that residuals did not show inhomogeneity or violate independence. The final model was used to extract coefficients and intercepts for each session and to provide global statistical evaluations. *p*-values obtained from Mixed models applied to each electrode were Bonferroni corrected.

This model provided data, including intercept values at different levels of PFB, for the BL period analysis in Fig 2D–2F. We then wished to understand how well this model explained beta power during the MPTP period. We tested data from each full week of the protocol (BL and then MPTP periods) to the selected model in an iterative fashion. This allowed us to extract fixed effects at each point in the protocol, generate a measure of week-to-week variability during BL, and then study the continued influence of those fixed effects as the dopamine lesion developed. So at each step in the MPTP period, we tested whether beta power was still being modulated as it had been in the BL period. To do this, we tested fixed effects using the Wald conditioned F test, and we illustrate the dynamic over the lesion using the model coefficient for each factor, presented in the figures (Figs 5 and 7). Where the effect is significant in the model, the line is solid (for example, throughout Figs 5C and 7B). Where the effect is not significant, we still report the coefficient but on a dotted line (Fig 7C). In this approach, we make the assumption that we can treat all of the recording sessions in a given week or at a given dose level as being a repeated measure, modeled as a random effect in the lme model. We confirmed that these random factors were indeed contributing to the model by comparing a model with and without them at every step. In every case, the random factors significantly improved the fit of the model.

As a final analysis of these factors during the MPTP period, we constructed a model that incorporated the whole MPTP period, and not just individual weeks. We used this to test for the influence on beta power of acute effects of the MPTP injection, by including a further factor of “days since MPTP injection.” This factor would account for modulation of beta after injection, which is subsequently compensated for. In applying the same model selection procedure as above, however, this factor was not retained in the selected model, and so there was no acute compensation effect to report.

We applied all of these procedures to models using the PFB factor to code behaviour—a factor describing the feedback previously received. However, we also confirmed the coding of cognitive control in beta oscillations by applying the same procedures as above to models coding behaviour separately in terms of a phase factor (SEA and REP) and an outcome factor on that trial (Correct and Incorrect).

## Supporting Information

S1 ARRIVE Checklist ARRIVE Checklist completed for this study. (PDF)

S1 Fig PST task example and ECoG implants. A. A sample time-course for a problem sequence in PST4. In the first problem, the monkey makes one incorrect response (INC) before finding the correct target (COR), and together these two trials are the SEA phase. The monkey then repeats this COR response three further times, completing the REP phase. An STC is then presented, which tells the monkey to restart the SEA. In the second SEA, the monkey makes two INC before finding the COR. After completing a large and fixed number of these problems, the monkey sees a large salient green circle on the screen, announcing the delivery of the large final bonus reward. B. ECoG implants of the two monkeys, with stereotaxic positions of the trans-cranial electrodes. Yellow and white dots represent the electrode positions projected onto a 2D stereotaxic grid in millimeters. The grid used for the current study is represented by the yellow dots only. Underlying this grid is a standard line drawing of the vertical view of monkey frontal surface anatomy for reference. Pink dots indicate the location of the reference electrode buried in the bone of the brow.
(TIF)

S2 Fig Equivalent of Fig 1D, but for reaction times (GO signal to lever release). As for response times, there is a significant difference for between high- and low-control trials, albeit one that is only marginally significant for Monkey R.
(TIF)

S3 Fig Analysis of FRPs on search (SEA) trials, to assess the impact of outcome probability on effects. A. Difference waves in BL generated in the same way as Fig 2B, but here for the difference INC-CO1 (solid lines), and CO1-COR (dotted lines), where CO1 is the 1st correct feedback in each problem. B. Proportions of INC trials for the first three trials of SEA for the whole BL, demonstrating similar proportions of INC and CO1 trials overall in the analysis shown in (A). C. Evolution of the difference peak (INC-CO1) of FRPs during the MPTP period. This figure is the exact equivalent of Fig 4A but for CO1 instead of COR. As before, significant difference emerges at full dose only for Monkey R. This effect is not significant for Monkey S, albeit there is no significant difference between INC and CO1 at full dose for this animal.
(TIF)

S4 Fig Binding of DAT as revealed by [^11^C]PE2I-BP_ND_ from PET imaging for the BL and then successive scans during the MPTP period. Each point represents the BP_ND_ measured for all voxels inside the Caudate, Putamen, and ACC cingulate ROIs as defined by Ballanger et al [57]. These data replicate the previous finding of Vezoli et al. [53] by showing an early pre-symptomatic striatal increase in BP_ND_ relative to baseline, followed by a slow reduction as the lesion progresses. The cingulate ROI has significantly lower BP_ND_, but shows a similar pattern. Note that [^11^C]PE2I-BP_ND_ is a specific measure of the presence of the dopamine transporter and cannot be considered as a direct index of dopamine levels.
(TIF)

## Abbreviations

BL: baseline
COR: correct
DA: dopamine
DAT: dopamine active transporter
ECoG: electrocorticography
EEG: electroencephalography
ERN: error-related negativity
FRP: feedback-related potential
INC: incorrect
LFP: local field potential
MCC: midcingulate cortex
MPTP: 1-methyl-4-phenyl-1,2,3,6-tetrahydropyridine
PD: Parkinson’s disease
PFC: prefrontal cortex
PMRS: Parkinsonian Monkey Rating Scale
PST: problem-solving task
REP: repetition phase
ROI: region of interest
RT: response time
SEA: search phase
STC: signal to change
TH: tyrosine hydroxylase
