# Supplementary figures and images for: Prefrontal Markers and Cognitive Performance Are Dissociated during Progressive Dopamine Lesion
Source: PLoS Biol. 2016 Nov 8;14(11):e1002576. doi: 10.1371/journal.pbio.1002576 (PMC5100991; doi:10.1371/journal.pbio.1002576)

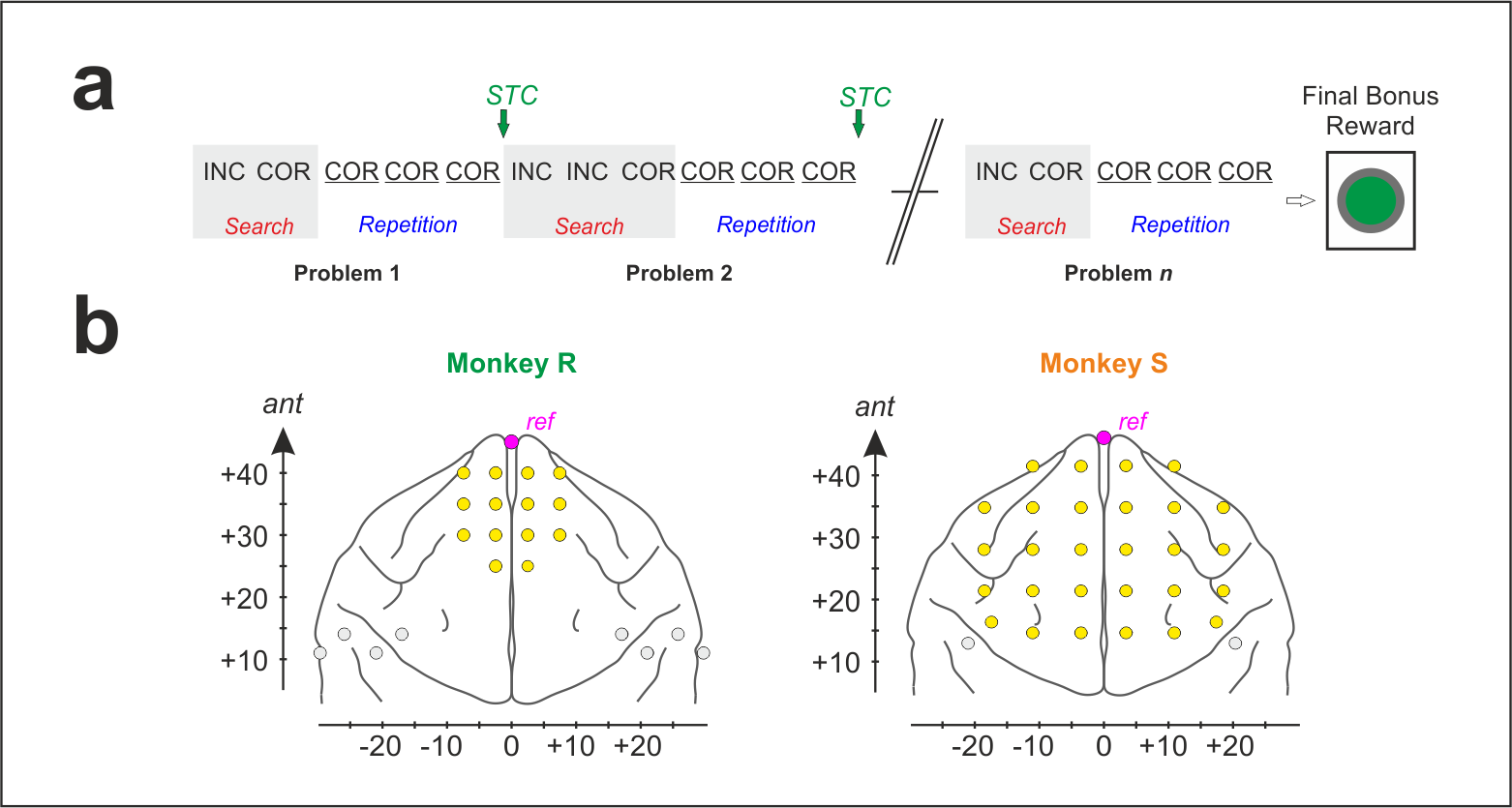

Supplement: S1 Fig — A. A sample time-course for a problem sequence in PST4. In the first problem, the monkey makes one incorrect response (INC) before finding the correct target (COR), and together these two trials are the SEA phase. The monkey then repeats this COR response three further times, completing the REP phase. An STC is then presented, which tells the monkey to restart the SEA. In the second SEA, the monkey makes two INC before finding the COR. After completing a large and fixed number of these problems, the monkey sees a large salient green circle on the screen, announcing the delivery of the large final bonus reward. B. ECoG implants of the two monkeys, with stereotaxic positions of the trans-cranial electrodes. Yellow and white dots represent the electrode positions projected onto a 2D stereotaxic grid in millimeters. The grid used for the current study is represented by the yellow dots only. Underlying this grid is a standard line drawing of the vertical view of monkey frontal surface anatomy for reference. Pink dots indicate the location of the reference electrode buried in the bone of the brow. (TIF) [file pbio.1002576.s002.tif]

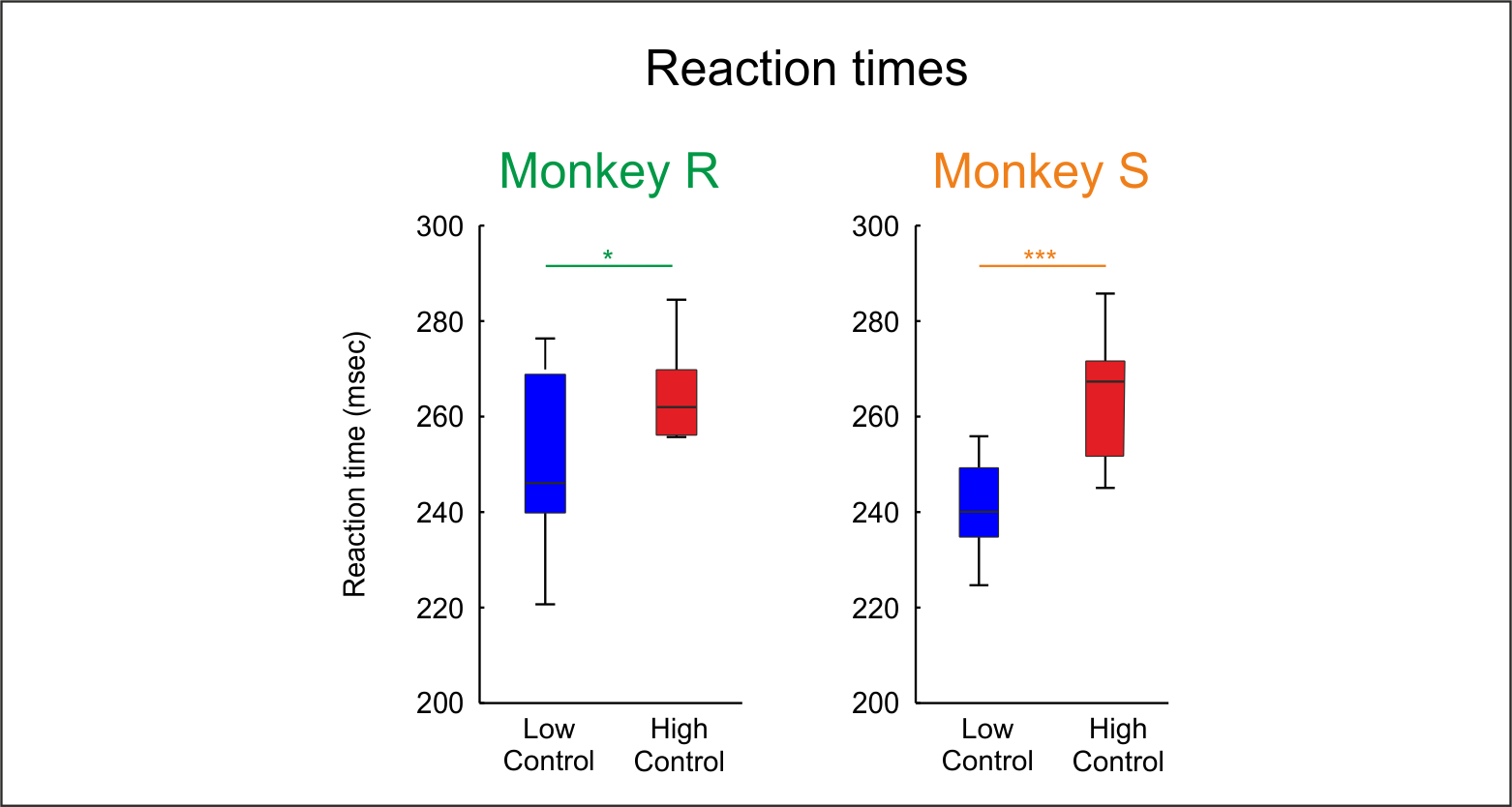

Supplement: S2 Fig — As for response times, there is a significant difference for between high- and low-control trials, albeit one that is only marginally significant for Monkey R. (TIF) [file pbio.1002576.s003.tif]

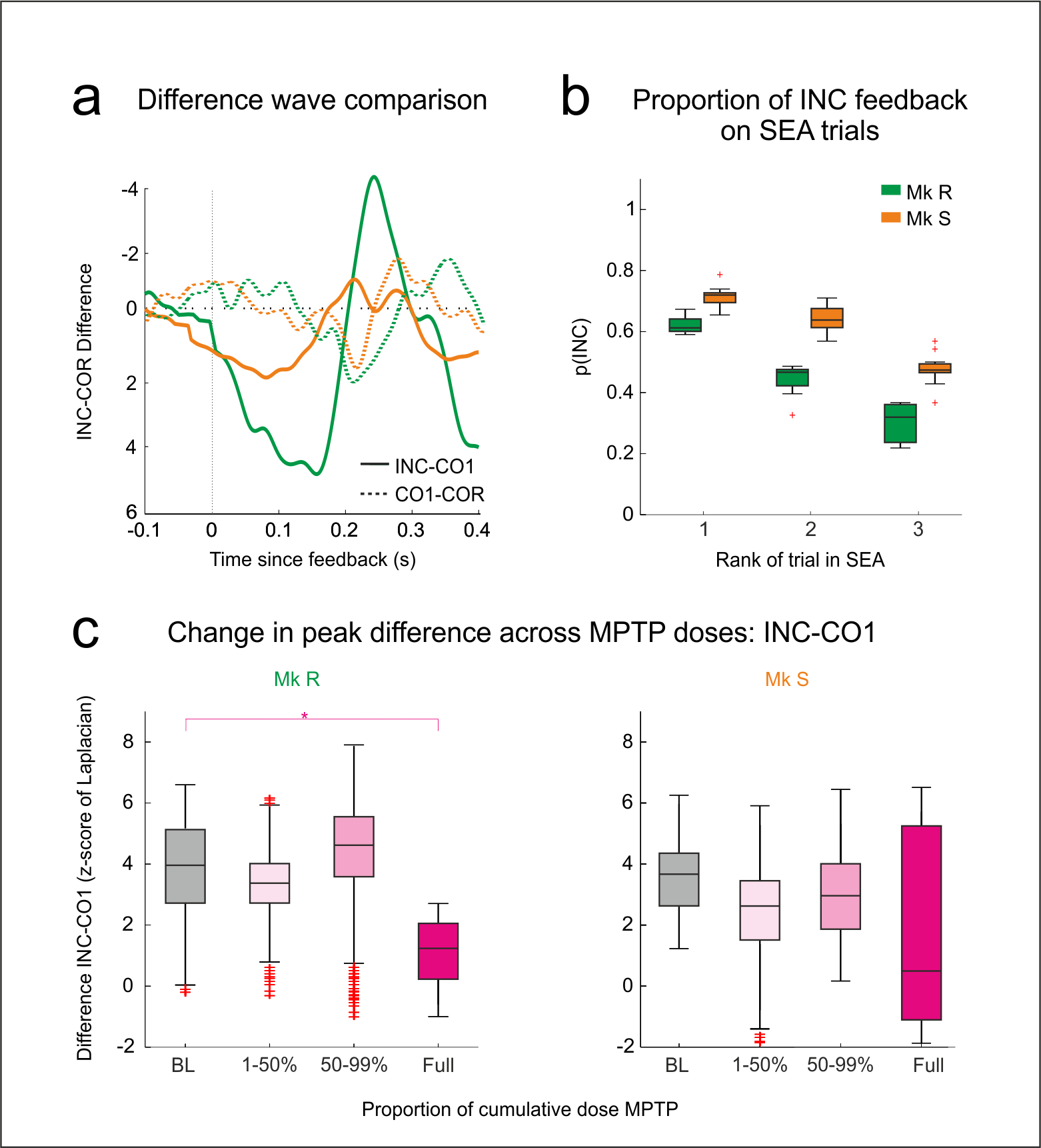

Supplement: S3 Fig — A. Difference waves in BL generated in the same way as Fig 2B, but here for the difference INC-CO1 (solid lines), and CO1-COR (dotted lines), where CO1 is the 1st correct feedback in each problem. B. Proportions of INC trials for the first three trials of SEA for the whole BL, demonstrating similar proportions of INC and CO1 trials overall in the analysis shown in (A). C. Evolution of the difference peak (INC-CO1) of FRPs during the MPTP period. This figure is the exact equivalent of Fig 4A but for CO1 instead of COR. As before, significant difference emerges at full dose only for Monkey R. This effect is not significant for Monkey S, albeit there is no significant difference between INC and CO1 at full dose for this animal. (TIF) [file pbio.1002576.s004.tif]

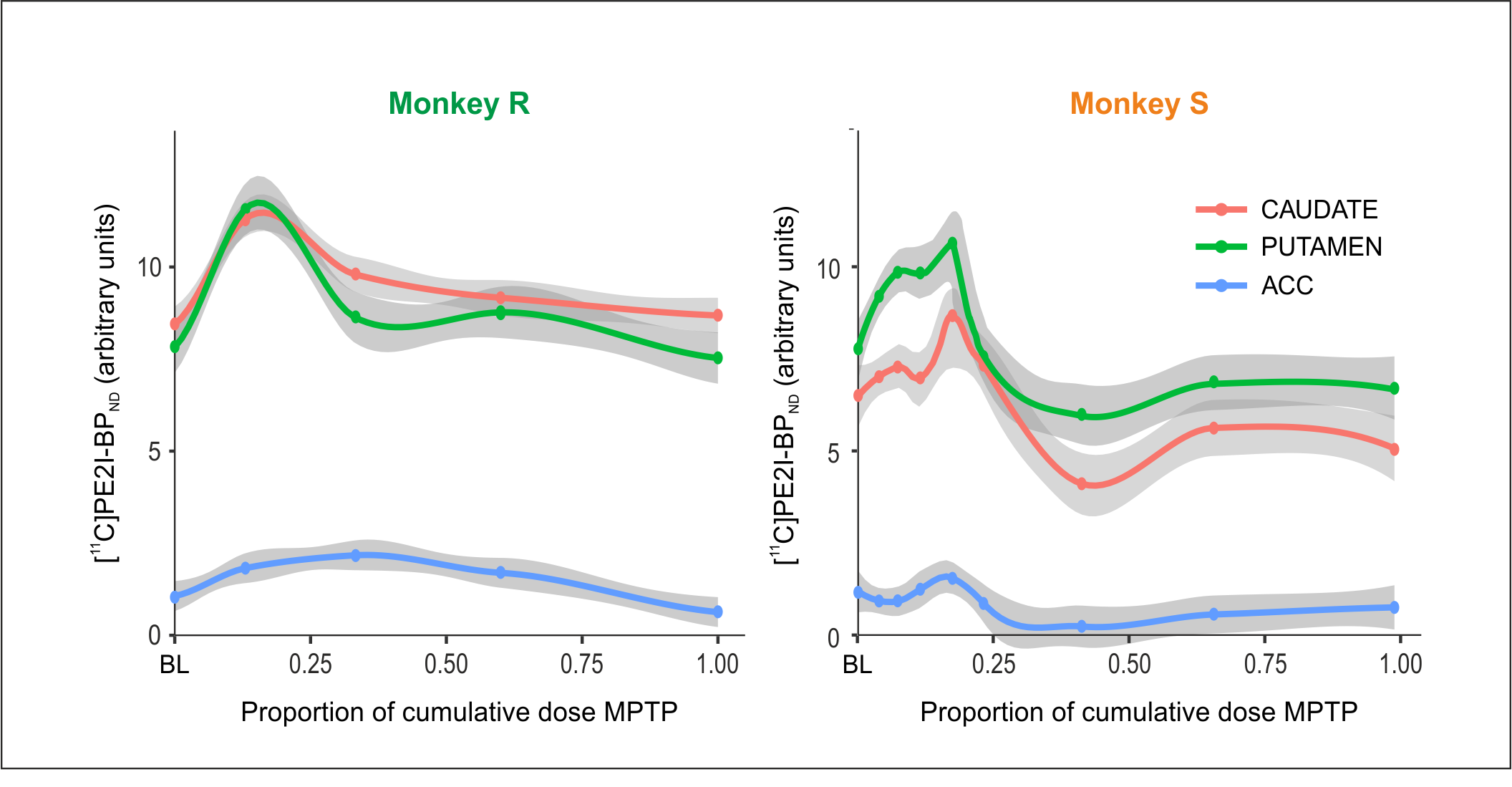

Supplement: S4 Fig — Each point represents the BPND measured for all voxels inside the Caudate, Putamen, and ACC cingulate ROIs as defined by Ballanger et al [57]. These data replicate the previous finding of Vezoli et al. [53] by showing an early pre-symptomatic striatal increase in BPND relative to baseline, followed by a slow reduction as the lesion progresses. The cingulate ROI has significantly lower BPND, but shows a similar pattern. Note that [11C]PE2I-BPND is a specific measure of the presence of the dopamine transporter and cannot be considered as a direct index of dopamine levels. (TIF) [file pbio.1002576.s005.tif]
